# Relationship between mitochondrial DNA Copy Number and SIRT1 Expression in Porcine Oocytes

**DOI:** 10.1371/journal.pone.0094488

**Published:** 2014-04-18

**Authors:** Daichi Sato, Nobuhiko Itami, Hidetaka Tasaki, Shun Takeo, Takehito Kuwayama, Hisataka Iwata

**Affiliations:** Tokyo University of Agriculture, Funako, Atugi City, Japan; RIKEN Advanced Science Institute, Japan

## Abstract

The present study assessed the effect of resveratrol on the expression of SIRT1 and mitochondrial quality and quantity in porcine oocytes. Supplementing the maturation medium with 20 µM resveratrol increased the expression of SIRT1, and enhanced mitochondrial functions, as observed from the increased ATP content and mitochondrial membrane potential. Addition of resveratrol also improved the ability of oocytes to develop into the blastocyst stage following activation. The effects of resveratrol on mitochondrial number were examined by comparing the mitochondrial DNA copy number (Mt number) between group of oocytes collected from the same donor gilt ovaries. Supplementing the maturation medium with only resveratrol did not affect the Mt number in the oocytes. However, supplementing the maturation medium with 10 µM MG132, a proteasome inhibitor, significantly increased the amount of ubiquitinated proteins and Mt number by 12 and 14%, respectively. In addition, when resveratrol was added to the medium containing MG132, the Mt number increased significantly by 39%, this effect was diminished by the addition of the SIRT1 inhibitor EX527. Furthermore, supplementing the medium with MG132 and EX527 did not affect Mt number. The mean SIRT1 expression in 20 oocytes was significantly and positively correlated with the Mt number in oocytes collected from the same donor. This study suggests that the expression of SIRT1 is associated with the Mt number in oocytes. In addition, activation of SIRT1 by resveratrol enhances the biosynthesis and degradation of mitochondria in oocytes, thereby replenishing and improving mitochondrial function and the developmental ability of oocytes.

## Introduction

Mitochondria are multifunctional organelles that are important for energy production, apoptosis and calcium homeostasis. The mitochondrial number in oocytes is important for oocyte quality because this number is closely related to oocyte fertilization and developmental outcomes [1]–[3]. Mitochondrial number can be predicted by quantifying the mitochondrial DNA copy number (Mt number) using real time-PCR, based on reports that mitochondria in oocytes typically contain one copy of the mitochondrial genome [4]. The Mt-number in bovine oocytes increases during the growth of oocytes from early antral follicles to antral follicles *in vivo* and *in vitro* [5], [6], and the number remains the same or increases further during oocyte maturation [5], [7], [8]. However, little is known about how culture conditions affect Mt number in oocytes. The quality of mitochondria in cells is maintained by removing damaged mitochondria, followed by mitochondrial biogenesis and morphological changes including fission and fusion [9], [10]. It was suggested that the depolarization of mitochondria, ubiquitination of the mitofusin proteins Mfn1 and Mfn2 by Parkin, and their subsequent proteasomal degradation is required for the removal of damaged mitochondria by autophagy [11]–[14]. However, little is known about mitochondrial degradation in oocytes during *in vitro* maturation.

It is difficult to measure the Mt number at differential time points in the same oocyte, because measuring the Mt number inevitably damages the oocytes. In addition, the Mt number in oocytes varies greatly among donors, which can complicate the interpretation of data on the intrinsic effects of drugs on Mt number. We previously reported the kinetics of Mt number in bovine oocytes using a unique system where the predicted Mt number for a group of 10 oocytes closely resembled the Mt number of another group of 10 oocytes collected from the same donor [15]. Using this model, we can assess the effect of supplements in the maturation medium on the Mt number in oocytes.

Sirtuins are highly conserved class III histone deacetylases with homology to yeast silent information regulator 2 [16]. SIRT1 is the most studied sirtuin, and plays an important role in regulating the cell cycle, apoptosis, and other metabolic processes by interacting with several molecules including p53 and forkhead transcription factors [17]–[19]. In addition SIRT1 enhances the transcription of PGC-1α, Tfam, and NRF-1, which regulate mitochondrial biosynthesis [20], [21]. We recently demonstrated the beneficial effects of adding resveratrol to the in maturation medium on bovine oocyte quality [22]. Specifically, resveratrol enhanced ATP content and mitochondrial membrane potential (MMP), but did not affect Mt number in the oocytes. We therefore speculated that the similar Mt number observed in oocytes following resveratrol supplementation were due to the simultaneous generation and degradation of mitochondria during oocyte maturation. As such, inhibiting mitochondrial degradation should allow the effects of resveratrol on Mt number to be assessed. In the current study, we examined the effect of resveratrol added to the maturation medium on SIRT1 expression, the developmental ability of oocytes, and mitochondrial functions in porcine oocytes. We also evaluated the effect of resveratrol on Mt number by co-treating with the proteasome inhibitor MG132 and the SIRT1 inhibitor EX527, and we examined the relationship between SIRT1 expression and Mt number in oocytes. Results from this study reveal the relationship between SIRT1 and mitochondrial quality and quantity during oocyte maturation.

## Materials and Methods

### Chemicals and media

All the chemicals were purchased from Nacalai Tesque (Kyoto, Japan) unless otherwise indicated. The medium used for *in vitro* maturation (IVM) was North Carolina State University 37 solution [23], which contained 0.6 mM cysteine supplemented with folliclular fluid (10% v/v). Follicular fluids were collected from antrum follicles (3–6 mm in diameter), centrifuged (10000× *g* for 5 min) and stored at −30°C.

### Ovary collection

Ovaries from gilts were collected at a local slaughterhouse (Kanagawa Meat Center), placed in phosphate-buffered saline (PBS) containing 10 IU/mL of penicillin G potassium and 0.1 g/mL of streptomycin sulfate, and transported to the laboratory within 1 h. During the transport, the temperature of the ovaries was maintained at 37°C.

### *In vitro* maturation, activation and *in vitro* culture

During the 20 h maturation period, cumulus-oocyte complexes (COCs) were cultured in a maturation medium containing 1 mM dibutyryl cAMP (dbcAMP: Sigma Chemical Co., St Louis, USA), 10 IU/mL of equine chorionic gonadotropin (eCG, ASKA Pharma Co. Ltd, Tokyo, Japan), and 10 IU/mL of human chorionic gonadotropin (hCG, Fuji Pharma Co. Ltd, Tokyo, Japan). The oocytes were then transferred to maturation medium that lacked dbcAMP and the hormones, and were cultured for 24 h. Following IVM, oocytes were activated in a culture medium containing 10 µg/mL ionomycine, followed by culture in a medium containing 10 µg/mL cytochalasin B and cycloheximide for 6 h. After activation, the embryoss were cultured for 7 days in culture medium and the rate of blastulation and total blastocyst cell number were then examined. To determine the total blastocyst cell number, embryos were fixed in 4% paraformaldehyde, mounted on glass slides using an antifade reagent containing DAPI (ProLong gold antifade reagent with DAPI; Invitrogen, OR, USA), and observed using a fluorescence digital microscope (BZ-8000; Keyence, Tokyo, Japan). *In vitro* maturation was performed at 38.5°C in an atmosphere containing 5% CO_2_ and 95% air. *In vitro* embryo culturing was performed at 38.5°C in an atmosphere containing 5% O_2_, 5% CO_2_ and 90% N_2_.

### Measurement of the mitochondrial DNA copy number

The Mt number in immature (immediately after collection) and mature (after 44 h of culture) was determined by examining two groups of 10 oocytes each collected from the same donor. Oocytes were denuded from granulosa cells, and the DNA extraction and polymerase chain reaction (PCR) protocols were performed according to methods described in previous reports [15]. Mt number was determined by performing real-time PCR using a Rotor-Gene 6500 real-time rotary analyzer (Quiagen GmbH, Hilden, Germany) with the primer set (5′-CGAGAAAGCACTTTCCAAGG-3′ and 5′-CTAATTCGGGTGTTGGTGCT-3′) and MESA Blue (Bio-Rad Ssofast-TM EvaGreen Supermix; Hercules CA USA). The primers were designed using Primer3Plus (http://sourceforge.net/projects/primer3/) and porcine mitochondrion gene information (Accession number AF304202) to amplify a 151-base pair (bp) region from 8744-8314. The PCR reactions were performed with an initial denaturation at 95°C for 1 min, followed by 40 cycles at 95°C for 2 s and 56°C for 10 s. A standard curve was generated for each run using 10-fold serial dilutions representing the copy number of the external standard. The external standard was the PCR product of the corresponding gene cloned into a vector using the Zero Blunt TOPO PCR cloning kit (Invitrogen, Carlsbad, CA, USA), and the PCR product was sequenced for confirmation before use. The amplification efficiencies of all trials were >1.9.

### Detection of SIRT1 by fluorescence immunostaining

Immature and mature oocytes were denuded from granulosa cells, and SIRT1 in oocytes was detected as described previously [24]. The primary and secondary antibodies used for this procedure were rabbit polyclonal anti-SIRT1 (1∶500; Santa Cruz Biotechnology, Santa Cruz, CA) and fluorescein-conjugated goat anti-rabbit IgG (1∶1000; Cell Signaling Technology Inc., Beverly, MA), respectively. The oocytes were mounted on glass slides using an antifade reagent containing DAPI (ProLong gold antifade reagent with DAPI; Invitrogen, OR, USA), and were observed using a fluorescence digital microscope (BZ-8000; Keyence, Tokyo, Japan). Fluorescence images of the oocyte were captured, and the fluorescence intensity was measured using the ImageJ software (BZ-8000; Keyence, Tokyo, Japan). To validate the immunostaining, the oocytes were cultured with the primary antibody (2 µg/mL IgG) or primary antibody and SIRT1 peptide (Abcam 7770-100, 2 or 10 µg/mL). As expected, the fluorescence intensity decreased significantly in a peptide concentration-dependent manner.

### ATP measurement

*In vitro* matured oocytes were denuded from the granulosa cells and the ATP content of oocytes was determined by measuring the luminescence generated in an ATP-dependent luciferin–luciferase bioluminescence assay (ATP assay kit; Toyo-Inc., Tokyo, Japan), as described previously [15]. Each sample was prepared by adding individual oocytes to 50 µL of distilled water.

### Mitochondrial membrane potential

*In vitro* matured oocytes were denuded from the granulosa cells and the incubated with MitoTracker Orange CMTMR (Invitrogen) for 30 min, then mounted onto glass slides for observation using a fluorescence digital microscope (BZ-8000; Keyence, Tokyo, Japan). The fluorescence intensity of the oocytes was measured using the ImageJ software (NIH, Bethesda, MD, USA).

### Western blot analysis

COCs were culture in medium containing 0 or 10 µM of MG132 and after maturation periods oocytes were denuded from the granulosa cells and subjected to western blot analysis. To quantify ubiquitinated protein, 40 oocytes were lysed in 20 µL of Laemmli sample buffer (Bio-Rad Laboratories Inc., Hercules, CA, USA) and analyzed by western blot, using a method described previously [25] with slight modifications. Briefly, rabbit polyclonal ubiquitin primary antibody (1∶1000; #3933; Cell Signaling Technology) was diluted using immunoreaction enhancer solution 1 (Toyobo, Osaka Japan), And donkey anti-rabbit IgG HRP-linked secondary antibody (1∶20 000; Abcam, Tokyo, Japan) was diluted in immunoreaction enhancer solution 2. The film obtained from the measurement was scanned using Alpha Imager Mini (Alpha Innotech Corporation, San Leandro, CA, USA), and the density of each lane was measured using Alpha View (Alpha Innotech Corporation).

### Experimental design

#### Experiment 1: Correlation between the Mt number of oocytes collected from the same donor

Twenty oocytes were collected from each donor and divided into two groups of 10 oocytes. With the 2 groups, the Mt number was measured, and the two values were then compared using 10 differential gilts (Figure S1). Correlations between the Mt numbers were calculated for both mature and immature oocytes.

#### Experiment 2: The effect of supplementing the maturation medium with resveratrol on SIRT1 expression levels and the developmental competence of oocytes

Oocytes were selected randomly from pooled oocytes derived from tens of gilts and were cultured in a medium containing 0 or 20 µM resveratrol. The concentration of resveratrol used was determined by a preliminary experiment, in which 20 µM of resveratrol was identified as the most effective concentration to enhanced the expression level of SIRT1 (Figure S2). After a maturation period, oocytes were then analyzed by immunostaining against SIRT1. The experiments were repeated three times using different sets of ovaries. The expression of SIRT1 in total of 88 oocytes matured in the control and resveratrol media were then compared. To assess the oocyte development, 30 *in vitro* matured oocytes that were cultured in a medium containing 0 or 20 µM resveratrol were activated, and the rate that oocytes progressed to the blastocyst stage and total cell number of observed blastocysts were determined. The experiment was repeated five times.

#### Experiment 3: Effect of resveratrol on mitochondrial function

Oocytes were selected randomly from pooled oocytes and cultured in IVM medium containing 0 µM or 20 µM resveratrol. The ATP content and MMP were then compared between the oocytes cultured in the presence and absence of resveratrol. The experiment was repeated three times using oocytes harvested from different ovaries.

#### Experiment 4: Effect of resveratrol on the Mt number in oocytes

To determine the effect of resveratrol on Mt number, oocytes were collected from individual gilts and divided into two groups as described Exp 1 (Figure S1). In each experiment, the two groups of oocytes were cultured as follows: Group 1, vehicle (DMSO 1∶1000) vs. resveratrol (20 µM); Group 2, vehicle (DMSO) vs. MG132 (10 µM); group 3, MG132 (10 µM) vs. MG132 (10 µM)+resveratrol (20 µM), Group 4, MG132 (10 µM) vs. MG132 (10 µM)+resveratrol (20 µM)+EX527 (20 µM), Group 5, vehicle (DMSO) vs. EX527 (20 µM); and Group 6, vehicle (DMSO) vs. EX527 (20 µM)+MG132 (10 µM). For these comparisons, 17, 20, 18, 20, 22, and 20 different donor gilts were used, respectively.

#### Experiment 5: Effect of MG132 on protein ubiquitination and oocyte mitochondrial function

We investigated whether culturing oocytes with MG132 inhibited the proteasomal degradation of ubiquitinated proteins and resulted in the accumulation of unhealthy mitochondria. Oocytes were cultured in a medium containing 0 or 10 µM MG132. After IVM, the levels of ubiquitinated proteins and ATP content were determined as described above. The experiments were repeated four and three times by using different sets of ovaries, respectively.

#### Experiment 6: Correlation between SIRT1 expression and Mt number in oocytes

We next examined the correlation between SIRT1 expression and Mt number in oocytes. More than 30 immature oocytes were collected from individual 24 gilts. Ten oocytes were used to measure the Mt number, and the rest were used for examining the level of SIRT1 expression by immunostaining. The correlation between Mt number and mean SIRT1 expression in oocytes was then calculated.

### Statistical analysis

Mean differences were compared using Student's *t*-test. Pearson's correlation was calculated using IBM SPSS ver. 21 (Statistical Package for the Social Sciences, SPSS Inc., Chicago, IL). A P value of <0.05 was considered to be statistically significant.

## Results

### Mt numbers from two distinct groups of 10 oocytes collected from the same gilt show high correlation

There was a significant correlation between the 2 Mt numbers in both immature and mature oocytes, (A, immature oocytes, r = 0.90; P<0.01 and B, matured oocytes, r = 0.85; P<0.01, Figure S3).

### Resveratrol enhanced the SIRT1 expression and improved the developmental ability of oocytes

**Figure 1:**
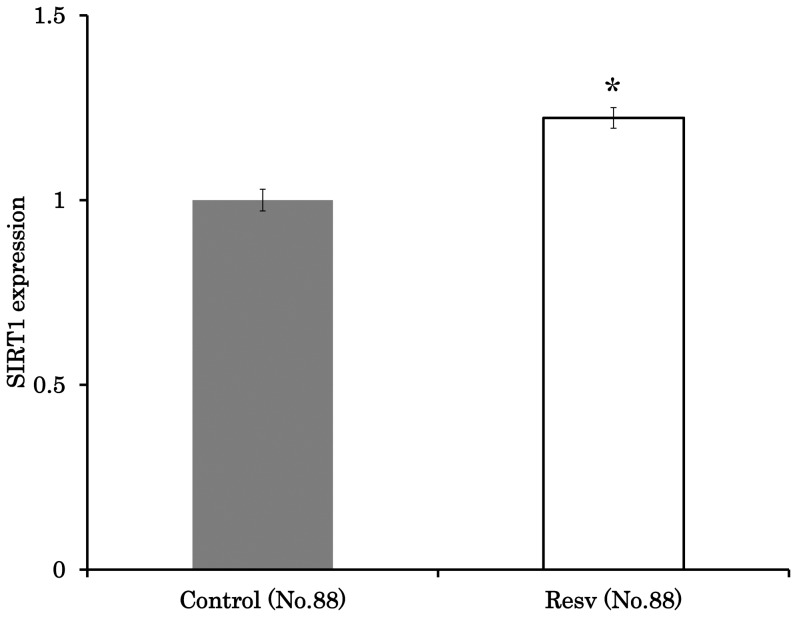
Effect of resveratrol on SIRT1 expression in oocytes. Comparison of SIRT1 expression in oocytes treated with 0 µM or 20 µM resveratrol. Average fluorescence intensity data were normalized to the value of 1 for controls. *,Letters indicate a significant difference (P<0.05).

Supplementing the maturation medium with resveratrol significantly enhanced the expression of SIRT1 (Figure 1). Resveratrol also improved the developmental ability of oocytes to the extent that the rate of blastulation and total blastocyst cell number were 16.7% and 62.8, respectively, which were significantly higher than that observed in control (rate of blastulation 9.3% and total cell number 51.1, P<0.05, Table 1).

**Table 1:** Effect of resveratrol on developmental ability of oocytes.

Resveratrol (µM)	No. of oocytes	No. of trials	Rate (%) of blastulation	Total cell number
0	150	5	9.3±1.1	51.5±2.8
20	150	5	16.7±2.1*	62.8±2.1*

Data are presented as mean ± SE.

*, P<0.05.

### Resveratrol improves oocyte mitochondrial function

**Figure 2:**
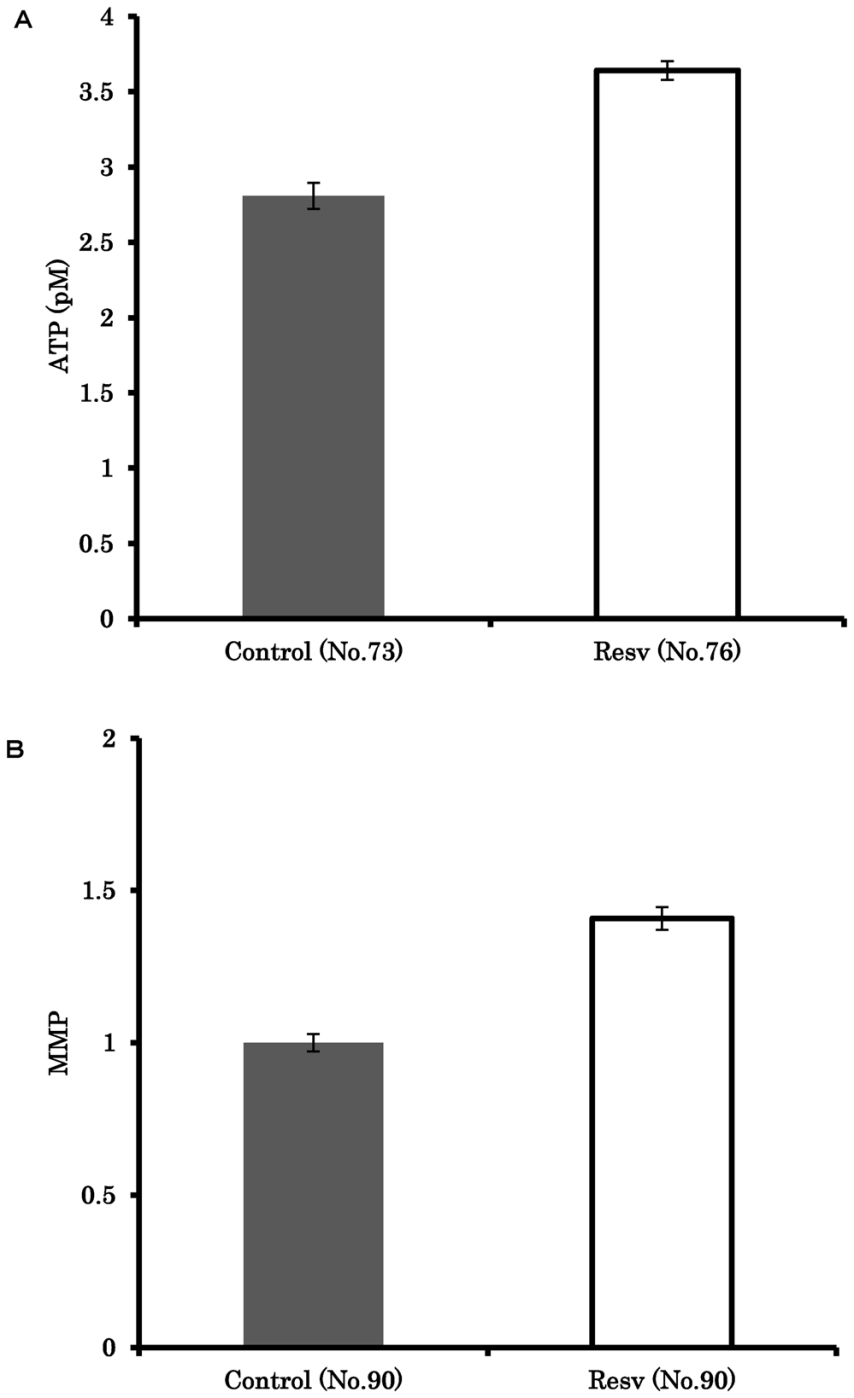
The effect of resveratrol on mitochondrial function in oocyte. Oocytes were matured in medium containing 0 or 20 µM resveratrol, and ATP (A) and mitochondrial membrane potential (B) were measured. The average fluorescence intensity data for MMP was normalized to the value of 1 for controls. *, Letters indicate a significant difference (P<0.05).

Supplementing the maturation medium with resveratrol significantly increased the ATP content compared to control (3.6±0.1 vs. 2.8±0.1, P<0.001; Figure 2A). The presence of resveratrol also significantly increased MMP of *in vitro* matured oocytes by 1.41-fold compared to controls (P<0.05; Figure 2B).

### Resveratrol enhances mitochondrial biosynthesis and degeneration

Mt numbers differed among individual gilts irrespective of the presence of resveratrol (Figure S4A), and the mean Mt numbers of donors were 287,573±23,620 for resveratrol groups and 296,839±22,161 for the control groups (Table 2 and Figure S4B). The relative Mt number of individual gilts is shown in Figure S4C and the mean of the relative Mt number is presented in Figure S4D, with the individual gilt data and mean Mt numbers of controls normalized to 1.0. To summarize the comparison of Mt number, results shown in Figures S4B and S4D are presented in Table 2.

**Table 2:** Effect of resveratrol, MG132 and EX527 on mitochondrial DNA copy number in oocytes.

Group	Supplement drugs	Number of donors	Mt number (Mean ± SE)	Relative
				Mt number (Mean ± SE)
1	Vehicle	17	296839±22161	1.00±0.00
	Resv	17	287573±23620	0.97±0.04
2	Vehicle	20	283948±14810	1.00±0.00
	MG132	20	315204±15134	1.14±0.06*
3	MG132	18	301838±33792	1.00±0.00
	MG132+Resv	18	357381±32728	1.39±0.17*
4	MG132	20	308347±16626	1.00±0.00
	MG132+Resv+EX527	20	312316±13562	1.07±0.08
5	Vehicle	22	279379±10984	1.00±0.00
	EX527	22	279060±9237	1.01±0.03
6	Vehicle	20	280948±17168	1.00±0.00
	MG132+EX527	20	249795±16379	1.05±0.18

Resveratrol (Resv), MG132, and EX527 was added at a concentration of 20 µM, 10 µM and 20 µM respectively.

*, P<0.05.

**Figure 3:**
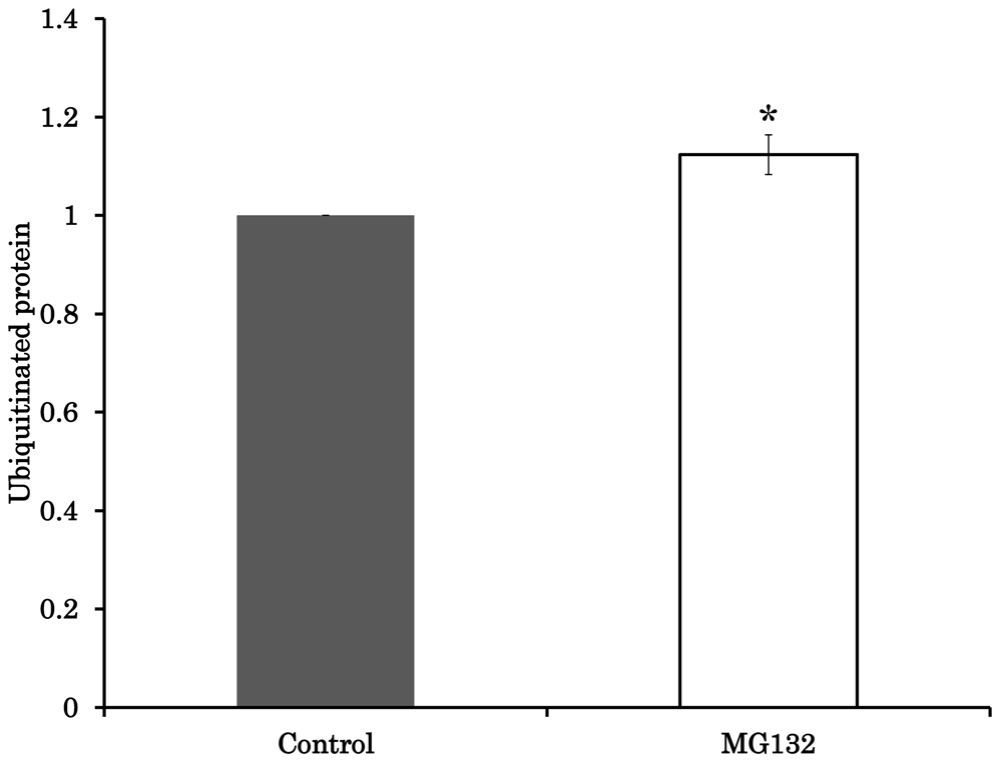
Effect of MG132 on amount of ubiquitinated protein in oocytes. Oocytes were cultured in medium containing 0 µM or 10 µM MG132. Comparison of the mean lane intensity between the two MG132 concentrations. Average intensity data were normalized to the value of 1 for controls. *,Letters indicate a significant difference (P<0.05).

Supplementing the maturation medium with the proteasome inhibitor MG132 increased the Mt number from 283,948 to 315,204, but this difference was not significant (P = 0.16). However, the relative Mt number of the MG132 group was significantly higher than 1.0 (114% vs. 100%; P<0.05; Group 2, Table 2). MG132 also significantly increased the amount of ubiquitinated proteins in the oocytes by 12% (Figure 3, Figure S5A). In addition, the ATP content of the oocytes was decreased by MG132 treatment compared to controls from 2.9 to 2.1 pM (Figure S5B, P<0.001). In contrast, the nuclear maturation was not significantly affected by MG132 (80.1±4.3 vs. 68.4±3.7; data not shown; P = 0.065). When oocytes were cultured with resveratrol and MG132, the Mt number was increased significantly by 39% (Group 3, Table 2, P<0.05), and this effect was inhibited by a SIRT1 inhibitor (EX527) so that Mt number did not differ between the MG132 and MG132+resveratrol+EX527 groups (Group 4, Table 2, P = 0.78). Furthermore, supplementation with EX527 or EX527+MG132 did not affect Mt number (Groups 5 and 6, respectively, Table 2).

### Mt number correlated with SIRT1 expression in oocytes

**Figure 4:**
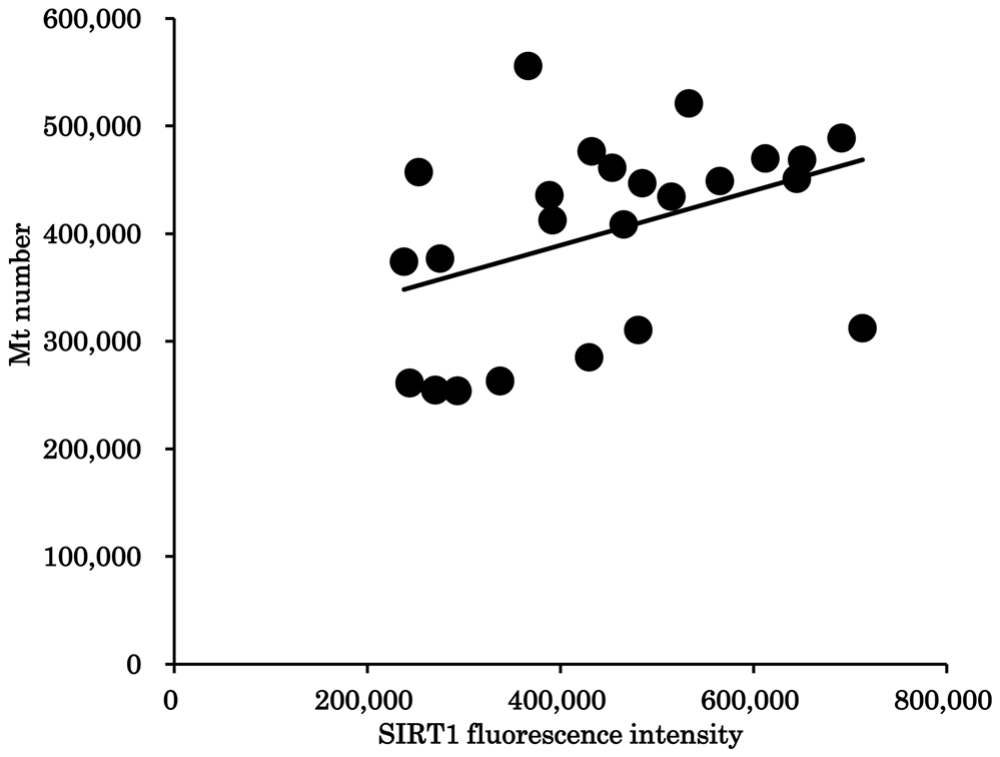
The relationship between SIRT1 expression and mitochondrial DNA copy number. The relationship between the mean SIRT1 expression and mitochondrial DNA copy number (Mt number) in cohort oocytes collected from the same gilt. The correlation coefficient is 0.413 (P<0.05).

The expression of SIRT1 in oocytes differed among individual gilts (Figure S6). SIRT1 expression was significantly and positively correlated with the Mt number of oocytes collected from the same donor (r = 0.413; P<0.05, Figure 4).

## Discussion

This investigation demonstrates for the first time that the addition of resveratrol to maturation medium upregulates the biosynthesis and degradation of mitochondria in porcine oocytes, which improves mitochondrial function and oocyte developmental competence.

The constant renewal of mitochondria is crucial for maintaining healthy mitochondria, and the number of mitochondria is continually balanced by cycles of generation and degeneration [26]. Although active mitochondrial biogenesis has been reported in oocytes [15], [27], little is known about mitochondrial homeostasis in these cells.

SIRT1 is a NAD+-dependent deacetylase that plays an important role in mitochondrial biogenesis and the degeneration of mitochondria via the process of mitophagy [26], [28]. Resveratrol is a well-known activator of SIRT1 that exerts beneficial effects on cellular mitochondrial function [29]–[31]. We previously reported that supplementing bovine oocyte maturation medium with resveratrol enhances the expression of SIRT1, the developmental ability of oocytes, and mitochondrial functions (as evidenced by the increase in the ATP content and MMP) [22], [24].

Consistent with previous reports on bovine oocytes [22], the current study demonstrates that resveratrol also enhances ATP content and MMP in porcine oocytes. Recently, Wang et al. [32] reported that supplementation of maturation medium improved oocyte maturation and further developmental ability by inducing progesterone secretion from the cumulus cells. They suggested that beneficial effect of resveratrol exerts through surrounding cells. However, we also found that culturing denuded oocytes with resveratrol significantly enhanced SIRT1 expression and ATP contents in oocyte (Figure S7A and B). From the results we suggest that resveratrol can directly affect mitochondrial function of oocyte, however effect of resveratrol on the surrounding cells remains elusive. In the previous study, oocytes were collected from six donor cows and cultured in a maturation medium containing 0 µM or 20 µM resveratrol. The Mt number differed among individual donors, and no significant difference in MT number was observed for the presence or absence of resveratrol. This investigation also showed that oocyte Mt number differed among individual gilts and that resveratrol had no effect on the number of mitochondria. Taken together, these studies raise the question how mitochondrial function improves with no changes in Mt number. Therefore, we hypothesized that mitochondrial turnover via biogenesis and degradation may complicate the precise monitoring of Mt number in oocytes. Interestingly, supplementing maturation media with MG132, a proteasomal inhibitor, significantly increased the relative Mt number in oocytes. Furthermore, the levels of ubiquitinated proteins increased and ATP content decreased. Consistent with this, a previous study showed that treating HeLa S3 cells with MG132 inhibited mitochondrial protein degradation but preserved mitochondrial mass [12]. Tanaka et al. [11] also reported that selective removal of mitochondria required the Pink-Parkin pathway, and that MG132 blocked the degradation of mitofusins on mitochondria that were depolarized by carbonyl cyanide 3-chlorophenylhydrazone treatment. Taken together, these data suggest that mitochondrial degradation occurs via the ubiquitin-proteasome degradation pathway during the meiotic maturation of porcine oocytes, and we suggest that unhealthy mitochondria accumulated when proteasomal-mediated mitochondrial degradation was inhibited by MG132.

**Figure 5:**
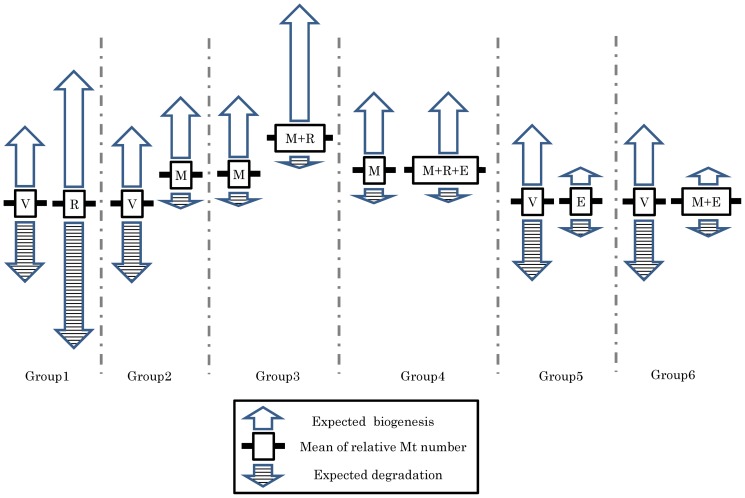
Summary of the effects of resveratrol, MG132, and EX527 on mitochondrial DNA copy number. Group 1: Supplementing the maturation medium with resveratrol did not affect mitochondrial DNA copy number (Mt number), which may be due to mitochondrial turnover via biogenesis and degradation. Group 2: MG132 significantly increased relative Mt number in oocytes by inhibiting mitochondrial degradation. Group 3: Supplementing media containing MG132 with resveratrol increased Mt number, which may be due to upregulated mitochondrial biogenesis. Group 4: Adding EX527 to the media containing MG132 diminished the effect of resveratrol on Mt number, suggesting that the upregulation of SIRT1 by resveratrol increased Mt number. Group 5 and Group 6: The addition of Ex527, an inhibitor of SIRT1, suppresses both mitochondrial generation and degeneration, which results in no difference between EX527 and vehicle or between EX527+MG132 and vehicle. V; Vehicle, R; Resveratrol, M; MG132, E; EX527, Bold line; mean of relative Mt number.

The molecular mechanism underlying the removal of mitochondria in oocytes, however, remains elusive, so we also examined the effect of resveratrol on Mt number while oocyte mitochondrial degradation was inhibited by MG132. As expected, resveratrol increased the relative Mt number by 39%, which suggests that resveratrol upregulates mitochondrial biosynthesis. In addition, a specific SIRT1 inhibitor (EX527) diminished the positive effect of resveratrol on Mt number in oocytes treated with MG132, suggesting that upregulation of SIRT1 by resveratrol plays a role in mitochondrial biogenesis. In this context, SIRT1 represses mTOR which in turn inhibits mitochondrial degradation by mitophagy [30], [33]–[35], and enhances the expression of PGC1α and upregulates mitochondrial generation [36]–[38]. Because supplementing the maturation medium with resveratrol alone had no effect on oocyte Mt number, we conclude that the upregulation of SIRT1 by resveratrol enhanced both mitochondrial biogenesis and mitochondrial degradation in porcine oocytes, thereby increasing mitochondrial turnover and improving mitochondrial function (Figure 5). Consistent with this idea, EX527 alone or EX527+MG132 had no effect on Mt number compared with vehicle controls, indicating that SIRT1 inhibition reduced mitochondrial biogenesis and degeneration.

In addition, Mt-number did not significantly change when EX527 or EX527+MG132 were supplemented to the medium, indicating that suppressing SIRT1 likely decreases mitochondrial biogenesis and degeneration. To further address the relationship between Mt number and SIRT1 expression in oocytes, we compared these parameters in cohorts of oocytes collected from the same donor gilt. There was a significant and positive correlation between SIRT1 expression and Mt number, suggesting either that high SIRT1 levels induce mitochondrial biosynthesis, or that large numbers of mitochondria in oocytes stimulate SIRT1 expression. Nevertheless, the relationship between cellular mitochondrial homeostasis and SIRT1 remains elusive.

The decline of mitochondrial function with age is a major focus of studies assessing age-associated subfertility [39]–[41], and it has been suggested that manipulating mitochondrial turnover might ameliorate age-associated cellular deterioration [39]. We previously reported the beneficial effects of resveratrol on the fertilization of oocytes collected from aged cows [24]. Based on results from this study, we suggest that the upregulation of SIRT1 with resveratrol can stimulate mitochondrial turnover in the oocytes of aged females, enhancing mitochondrial qualities such as ATP content and MMP and restoring the viability of oocytes that have been compromised due to age.

In conclusion, SIRT1 levels are closely related to Mt number in oocytes. Resveratrol upregulates SIRT1 expression and enhances mitochondrial function by upregulating mitochondrial biogenesis and degradation, resulting in improved oocyte development.

## Supporting Information

Figure S1 **Comparison of mitochondrial DNA copy number between two groups of oocytes derived from the same donor.** Twenty oocytes were collected from follicles (3–6 mm in diameter) of individual gilts (N = 10), and divided into 2 groups. Mt number was measured and compared between the two groups. Immature and matured oocytes were subjected to this comparison.
(TIFF)

Figure S2 **Effect of various concentration of resveratrol on the expression level of SIRT1.** Thirty oocytes were cultured in medium containing 0 µM, 2 µM, and 20 µM of resveratrol, and expression levels of SIRT1 were measured by immunostaining against SIRT1. Experiments were repeated three times. Average fluorescence intensity data were normalized to the value of 1 for controls.
(TIFF)

Figure S3 **Correlation between the 2 mitochondrial DNA copy numbers (Mt number) determined by groups of 10 oocytes from the same donor.** Twenty oocytes were collected from each donor and divided into two groups of 10 oocytes each. The mitochondrial DNA copy number (Mt number) was then measured in each group. Closed and open circle represent the Mt number determined from each oocyte group. The x-axis represents individual gilts, and the y-axis represents the Mt DNA copy numbers per oocyte. There was a significant correlation between the values obtained from each donor in both (A) immediately after oocyte collection and (B) after *in vitro* maturation (IVM; r = 0.90 and 0.85; P<0.01).
(TIF)

Figure S4 **Effect of resveratrol on mitochondrial DNA copy number in oocytes.** Two groups of 10 oocytes collected from 17 individual gilts were cultured in a medium containing 0 or 20 µM resveratrol. (A) Comparison of mitochondrial DNA copy number (Mt number) among individual gilts. (B) Comparison of mean Mt number between two resveratrol concentrations in all gilts. (C–D) Comparison of relative Mt number in oocytes; the Mt number of control oocytes was defined as 1. C, relative Mt number in each individual gilt. D, mean relative Mt number in all gilts.
(TIF)

Figure S5 **Effect of MG132 on amount of ubiquitinated protein in oocytes.** Oocytes were cultured in medium containing 0 or 10 µM MG132 and subjected to western blot against ubiquitin.
(TIF)

Figure S6 **SIRT1 expression in individual oocytes collected from each gilt.** Twenty oocytes were collected from individual gilts and subjected to immunestaining against SIRT1.
(TIFF)

Figure S7 **Effect of resveratrol on the expression level of SIRT1 and ATP content in denuded oocytes.** Denuded oocytes were cultured in maturation medium containing 0 or 20 µM of resveratrol and 0.9 mM pyruvate. Expression levels of SIRT1 and ATP content in oocytes were measured. Experiments were repeated three times. Expression of SIRT1 was examined by immunostaining. Average fluorescence intensity data were normalized to the value of 1 for controls.SIRT1.
(TIF)
