# Supplementary figures and images for: Relationship between mitochondrial DNA Copy Number and SIRT1 Expression in Porcine Oocytes
Source: PLoS One. 2014 Apr 18;9(4):e94488. doi: 10.1371/journal.pone.0094488 (PMC3991605; doi:10.1371/journal.pone.0094488)

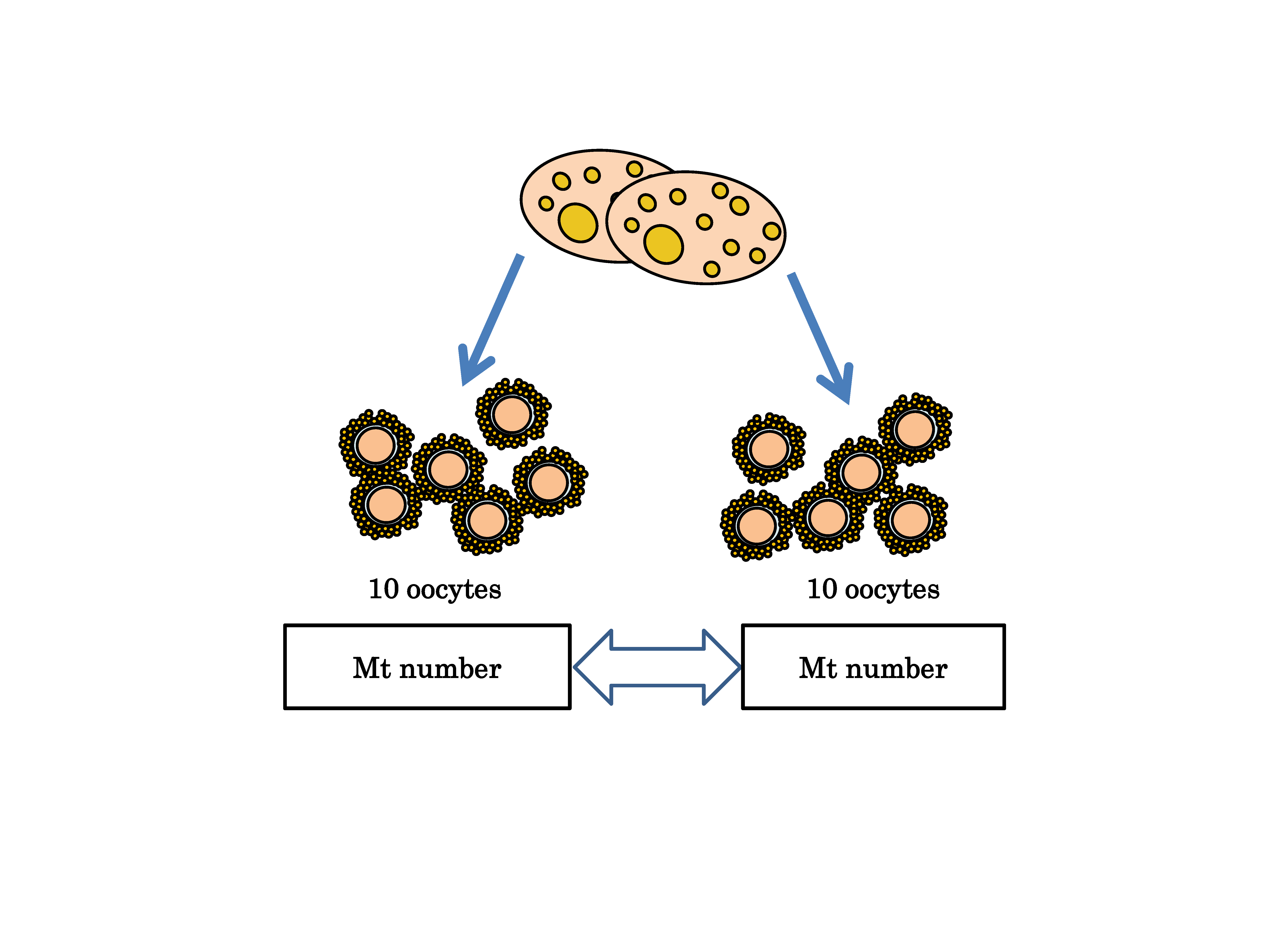

Supplement: Figure S1 — Comparison of mitochondrial DNA copy number between two groups of oocytes derived from the same donor. Twenty oocytes were collected from follicles (3–6 mm in diameter) of individual gilts (N = 10), and divided into 2 groups. Mt number was measured and compared between the two groups. Immature and matured oocytes were subjected to this comparison. (TIFF) [file pone.0094488.s001.tif]

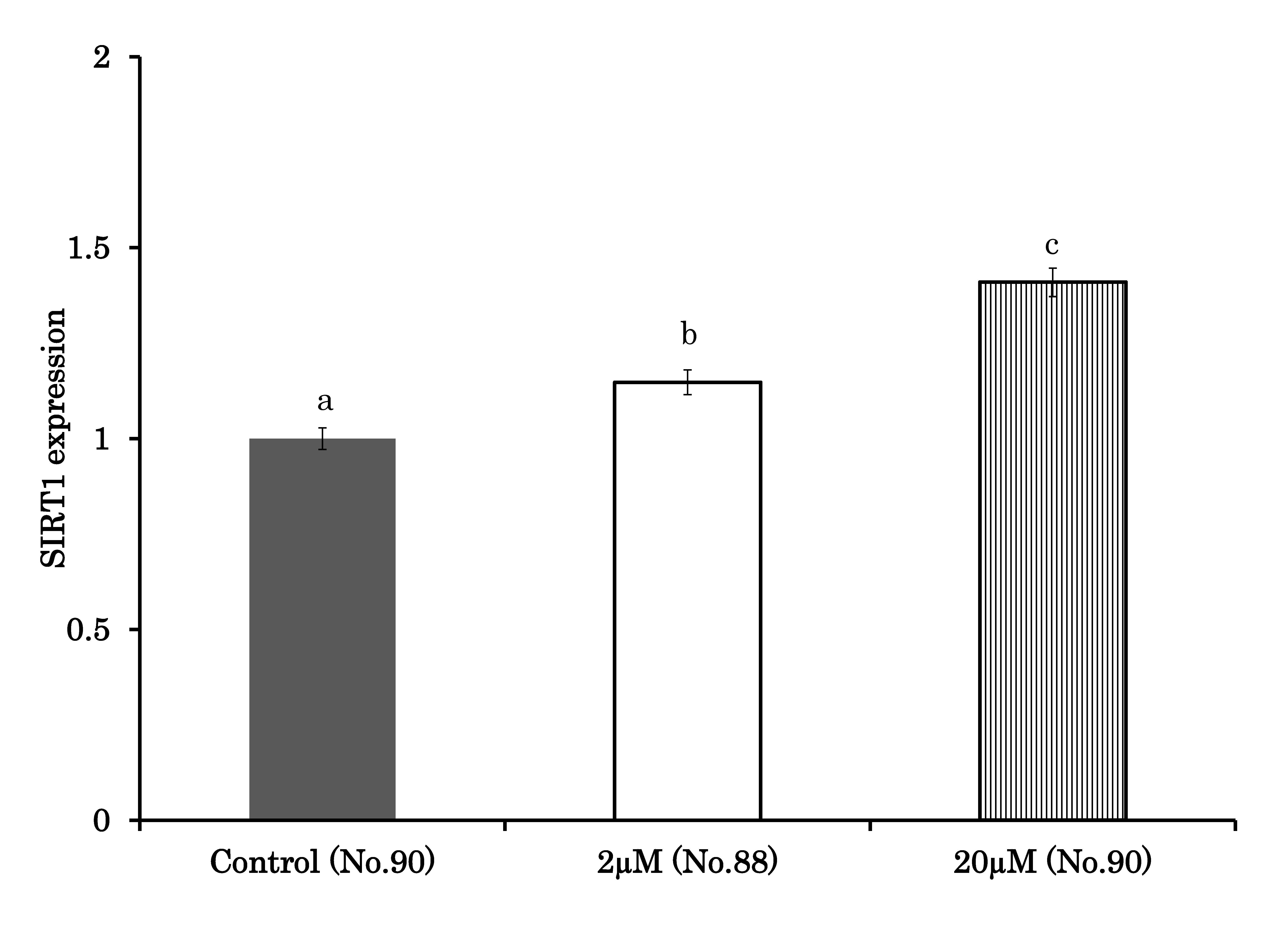

Supplement: Figure S2 — Effect of various concentration of resveratrol on the expression level of SIRT1. Thirty oocytes were cultured in medium containing 0 µM, 2 µM, and 20 µM of resveratrol, and expression levels of SIRT1 were measured by immunostaining against SIRT1. Experiments were repeated three times. Average fluorescence intensity data were normalized to the value of 1 for controls. (TIFF) [file pone.0094488.s002.tif]

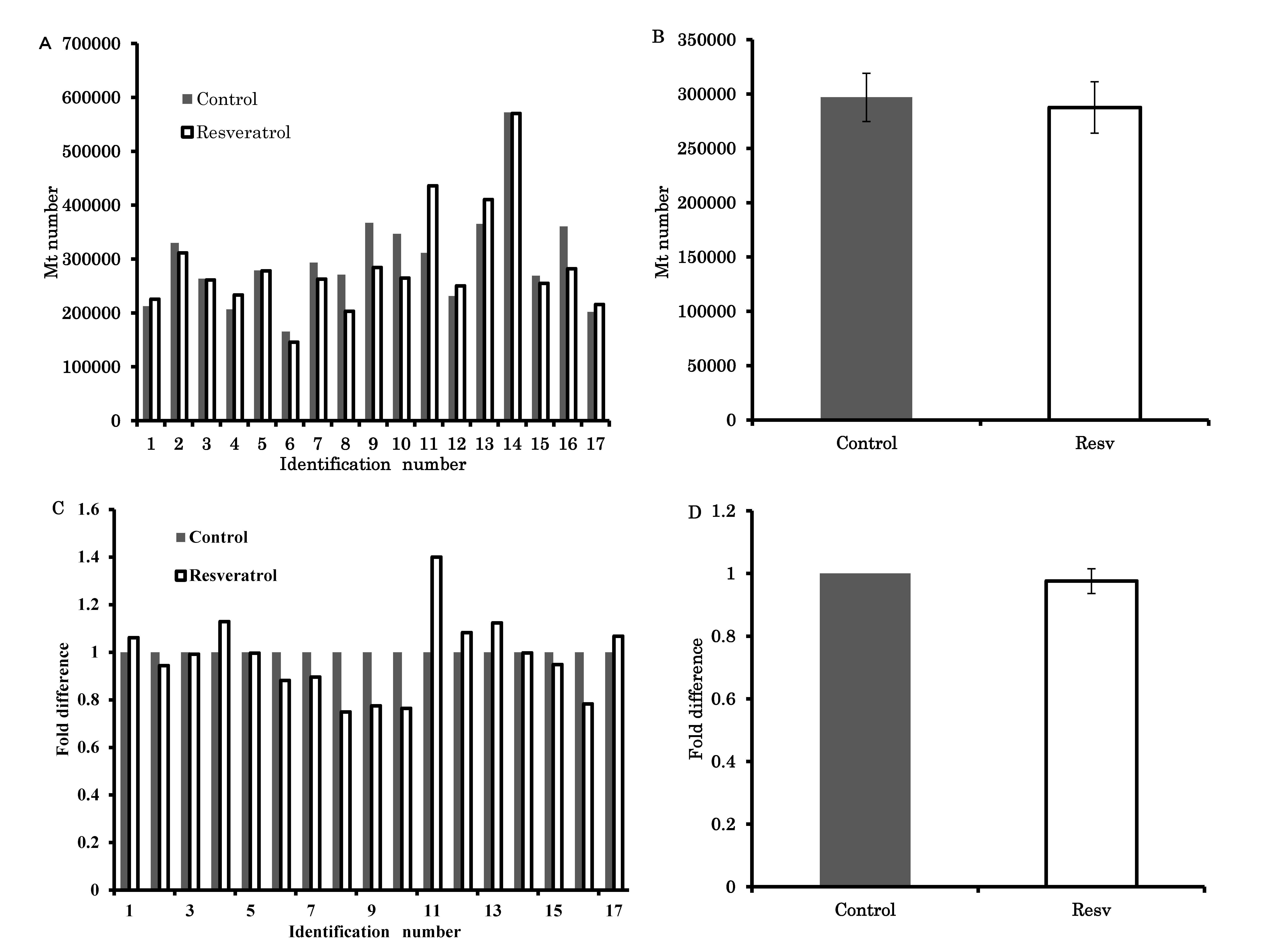

Supplement: Figure S4 — Effect of resveratrol on mitochondrial DNA copy number in oocytes. Two groups of 10 oocytes collected from 17 individual gilts were cultured in a medium containing 0 or 20 µM resveratrol. (A) Comparison of mitochondrial DNA copy number (Mt number) among individual gilts. (B) Comparison of mean Mt number between two resveratrol concentrations in all gilts. (C–D) Comparison of relative Mt number in oocytes; the Mt number of control oocytes was defined as 1. C, relative Mt number in each individual gilt. D, mean relative Mt number in all gilts. (TIF) [file pone.0094488.s004.tif]

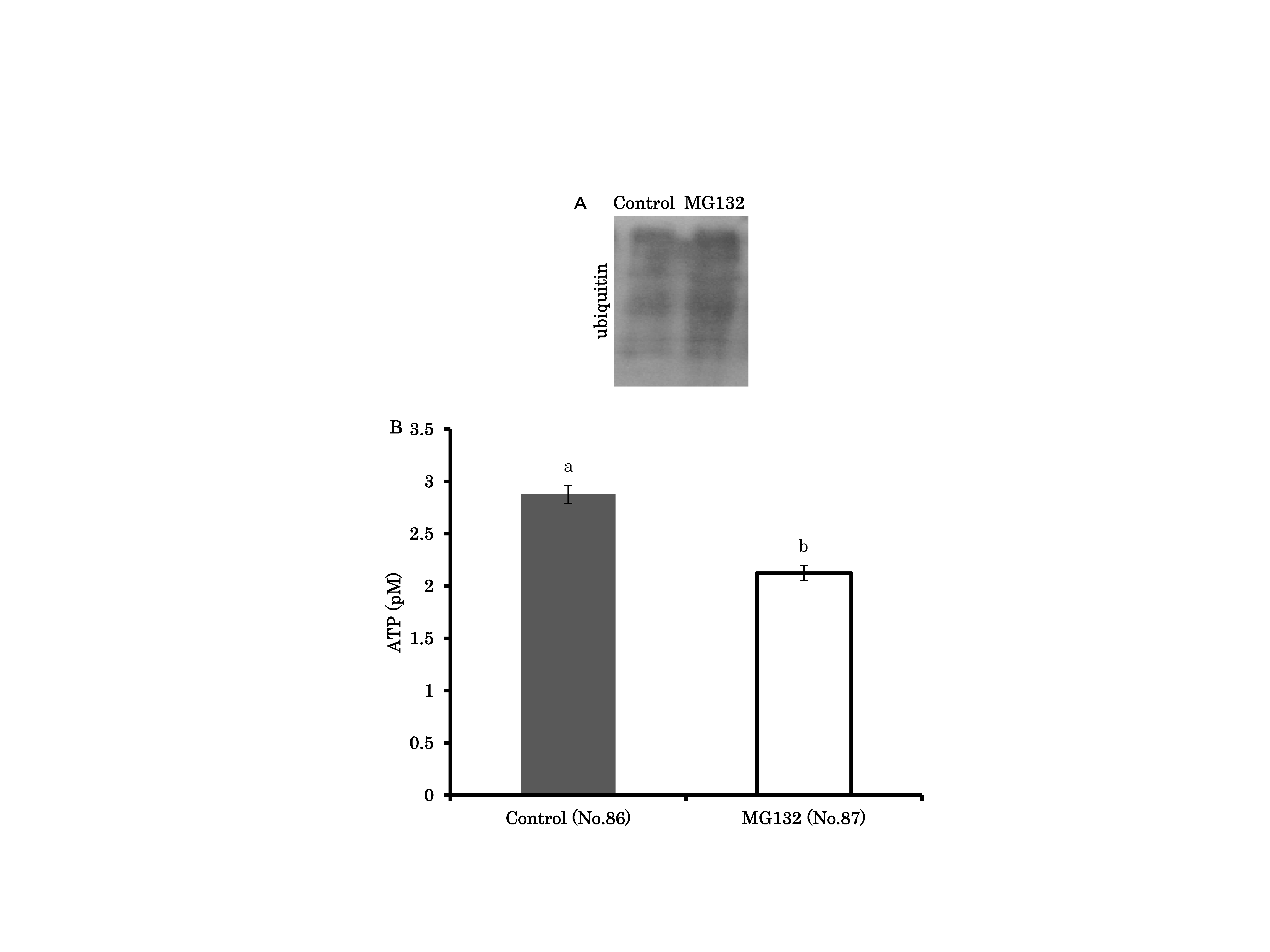

Supplement: Figure S5 — Effect of MG132 on amount of ubiquitinated protein in oocytes. Oocytes were cultured in medium containing 0 or 10 µM MG132 and subjected to western blot against ubiquitin. (TIF) [file pone.0094488.s005.tif]

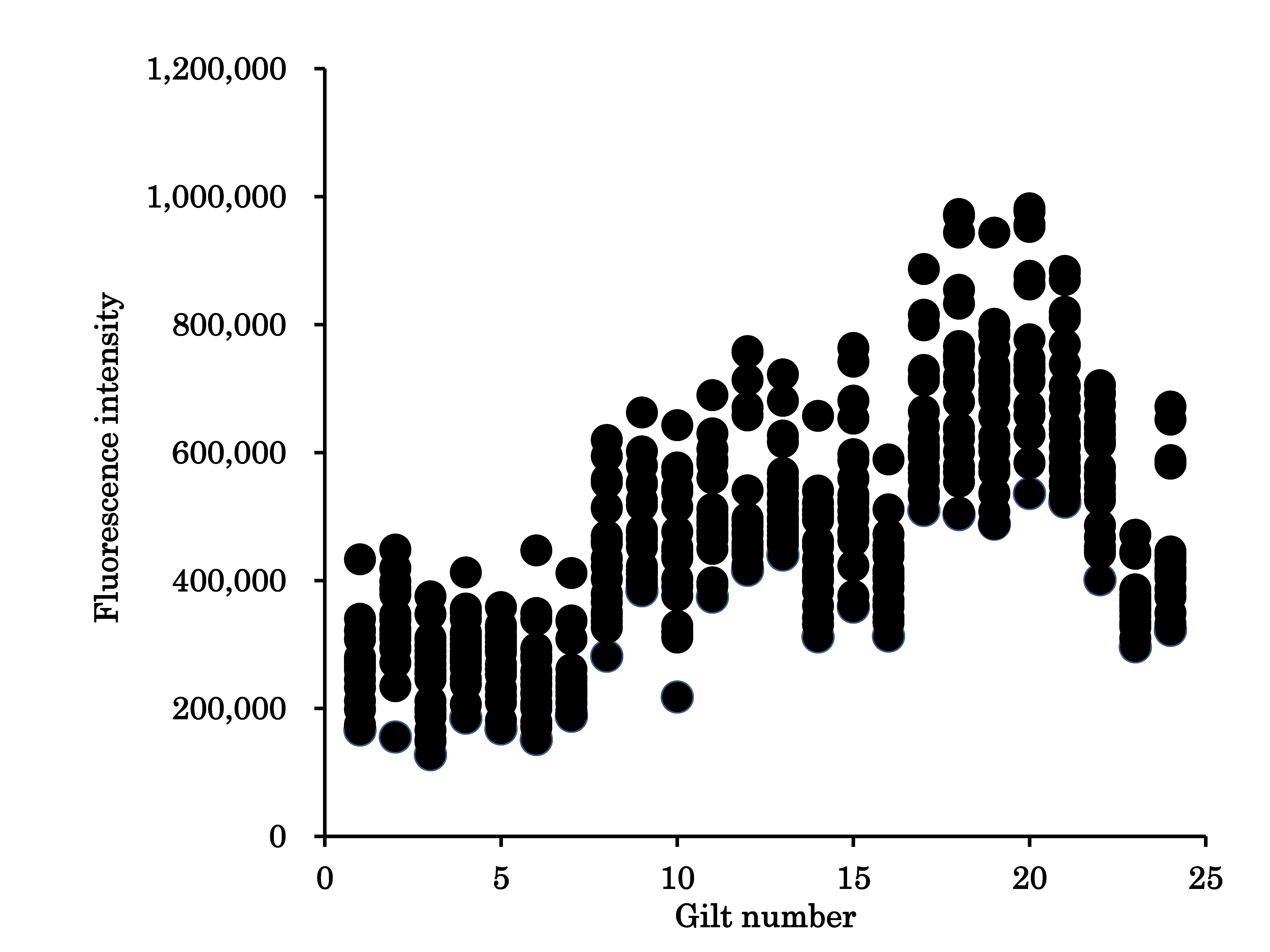

Supplement: Figure S6 — SIRT1 expression in individual oocytes collected from each gilt. Twenty oocytes were collected from individual gilts and subjected to immunestaining against SIRT1. (TIFF) [file pone.0094488.s006.tif]
